# Mapping the literature on improving primary-to-secondary care referrals through the lens of the Quintuple Aim framework for healthcare improvement: a scoping review

**DOI:** 10.1136/bmjopen-2025-105430

**Published:** 2026-08-03

**Authors:** Karl Oskar Björkman, Martin Rejler, Daniel Masterson, Eva Arvidsson, Sofi Fristedt

**Affiliations:** 1 School of Health and Welfare, Jönköping University, Jönköping, Sweden; 2 Bra Liv Vetlanda Primary Health Care Centre, Region Jönköping County, Vetlanda, Sweden; 3 Futurum Academy of Health and Care, Region Jönköping County, Jönköping, Sweden; 4 Department of Internal Medicine and Geriatrics, Höglandssjukhuset Eksjö, Region Jönköping County, Eksjö, Sweden; 5 Jönköping Academy for Improvement of Health and Welfare, School of Health and Welfare, Jönköping University, Jönköping, Sweden; 6 School of Health Sciences, University of Skövde, Skövde, Sweden; 7 Department of Health, Medicine and Caring Sciences, Linköping University, Linköping, Sweden; 8 Department of Health Sciences, Faculty of Medicine, Lund University, Lund, Sweden

**Keywords:** Primary Health Care, Quality Improvement, Review

## Abstract

**Abstract:**

**Objectives:**

To explore and map the literature on referral pathway gaps and improvement strategies in primary healthcare (PHC) to secondary healthcare (SHC) referrals, and to categorise both gaps and improvements using the Quintuple Aim framework for healthcare improvement—a model encompassing patient experience, population health, costs, provider well-being and health equity.

**Design:**

Scoping review guided by Arksey and O’Malley’s framework, refined by the Joanna Briggs Institute and reported following the Preferred Reporting Items for Systematic Review and Meta-Analysis extension for Scoping Reviews (PRISMA-ScR) guidelines.

**Data sources:**

PubMed, CINAHL and Scopus were searched for publications from January 2013 to May 2024.

**Eligibility criteria:**

Peer-reviewed, English-language studies reporting on improvements to PHC-to-SHC referral processes. Studies were eligible if they examined referrals initiated by primary care physicians (population) addressed interventions or improvements targeting referral gaps (concept) in PHC settings (context). Studies focused exclusively on secondary care perspectives, non-English publications and those outside the 2013–2024 date range were excluded.

**Data extraction and synthesis:**

Data were charted using a standardised form based on a Population–Concept–Context framework. Data extraction was partially supported by the artificial intelligence-based platform Elicit, with systematic verification against source articles. Referral gaps and improvements were mapped to the five Quintuple Aim domains using structured analytical columns applied to each included study, then synthesised narratively.

**Results:**

85 studies were included. Analysis revealed recurring referral pathway gaps across five stages (patient assessment, information transfer, coordination, specialty access and feedback), negatively impacting all five Quintuple Aim domains. Eight categories of improvements were identified (eg, educational initiatives, electronic referral systems, structured templates, decision support tools). These improvements frequently enhanced referral efficiency; however, evidence of impact on provider well-being and health equity was limited.

**Conclusions:**

Referral improvements can enhance efficiency, but evidence of impact on provider well-being and health equity remains sparse. Future research should prioritise equity-focused designs and address administrative burden and resource constraints to achieve full Quintuple Aim alignment.

**Trial registration number:**

Open Science Framework (https://osf.io/87htv/).

**STRENGTHS AND LIMITATIONS OF THIS STUDY**

- This review systematically mapped a wide range of international literature using a recognised scoping review methodology, including a comprehensive search strategy across three major databases and dual-reviewer screening for study selection.
- A key strength is the application of the Quintuple Aim framework, which provided a structured and multifaceted approach to analyse referral gaps and solutions across five critical domains of healthcare, leading to the identification of significant evidence gaps, particularly for provider well-being and health equity.
- As is characteristic of scoping reviews, this study did not undertake a formal quality appraisal or risk-of-bias assessment of the included articles; therefore, it does not assess the effectiveness of the identified referral solutions.
- The review was limited to English-language publications from 2013 to May 2024, and the use of the Quintuple Aim’s predefined domains may have meant that other relevant perspectives or conceptual nuances within the referral process were less prominent in the synthesis.
- Artificial intelligence (AI) was used to assist with parts of the data extraction process; while there was subsequent manual verification, the potential for AI-specific biases in study interpretation or charting cannot be entirely ruled out.

## Introduction

###  Background and rationale

Referral processes form a critical link between primary healthcare (PHC) and secondary healthcare (SHC), ensuring that patients receive timely, appropriate expertise when clinical needs exceed the resources or authority of their initial care provider. As healthcare systems grow more complex, effective referral pathways become essential for continuity of care, cost containment and patient satisfaction.^1 2^ PHC—often serving as the first point of contact—plays a crucial role in identifying when SHC input is necessary, thereby frequently acting as a gatekeeper to higher-level services.^3–6^

A conventional referral typically involves several steps: a PHC physician (eg, general practitioner) decides that referral is needed, composes and transfers pertinent patient information to the SHC, and then the patient attends an SHC appointment. Following evaluation or treatment, the SHC relays their findings and recommendations back to PHC, thereby enabling collaborative management.^7–10^

In practice, however, referral pathways are often undermined by systemic and physician-associated shortcomings—collectively referred to throughout this article as ‘gaps’. Some shortcomings reflect knowledge gaps, where key evidence on effective referral strategies is absent or incomplete, while others arise from knowledge–practice gaps, where proven practices are not consistently integrated into routine care. Examples of these gaps include incomplete, late or unnecessary referral letters, as well as fragmented coordination and follow-up systems. Still others reflect broader gaps in healthcare systems, such as insufficient attention to underserved populations.^11–13^ The gaps risk duplicating clinical investigations, prolonging wait times, heightening the potential for medical errors and eroding patient trust.^10 14–16^

Healthcare researchers and policymakers have long recognised the need for integrated frameworks that guide system-wide improvements. One widely known model is the Triple Aim framework, originally created to balance three interdependent goals: enhancing the patient experience of care, improving population health and reducing healthcare costs.^17^ Building on these objectives, the Quadruple Aim expanded the framework by adding an emphasis on provider well-being. Most recently, the Quintuple Aim emerged, incorporating health equity as a fifth focal area.^18 19^ By explicitly addressing social and structural determinants of health, the Quintuple Aim offers a comprehensive framework for examining referral processes—particularly in understanding how solutions can impact not only patients but also providers, healthcare systems and underserved communities. By applying the Quintuple Aim as a guiding framework, this review evaluates referral interventions through the interplay of quality, cost, provider well-being, population health and equity.

Existing systematic reviews have examined discrete aspects of the referral process, including integrated care models,^20^ elective surgery referrals,^21 22^ oncology coordination^23^ and referral letter quality.^24^ Other reviews have focused on electronic referral systems,^25^ single-entry intake models,^26^ continuity in cancer care^27 28^ and performance measurement,^29^ while studies by Blank,^15^ Greenwood-Lee,^10^ Faulkner^30^ and Imison and Naylor^31^ document interventions at various levels.

However, these studies often adopt a SHC-focused lens and do not map referral interventions from a PHC standpoint. Consequently, there is a need for a synthesis that (1) catalogues referral pathway gaps and improvements in PHC-to-SHC referrals, (2) examines the reported outcomes and (3) appraises their broader system impacts under the Quintuple Aim framework for healthcare improvement. A search of the Open Science Framework and PROSPERO, conducted during the development of this review’s protocol,^32^ confirmed that, at that point of time, no previously registered protocols had addressed PHC-to-SHC referral improvements from a Quintuple Aim perspective.

### Objective

The objective of this scoping review is to explore and map the literature on referral pathway gaps and improvement strategies in PHC-to-SHC referrals, and to categorise both gaps and improvements by applying the Quintuple Aim framework for healthcare improvement.

### Research questions

1. What referral pathway gaps have been identified in PHC-to-SHC referrals, and which improvements have been studied to address them?
2. What outcomes have been reported for these improvements?
3. How do these improvements align with the Quintuple Aim of healthcare improvement?

## Methods

This scoping review was guided by the five-stage framework outlined by Arksey and O’Malley, further refined by the Joanna Briggs Institute and reported following the Preferred Reporting Items for Systematic Review and Meta-Analysis extension for Scoping Reviews (PRISMA-ScR) guidelines^33–35^ (checklist in online supplemental file 1). The methodology involved six clearly defined stages: (1) defining the research questions, (2) identifying relevant studies, (3) selecting studies, (4) charting data, (5) collating, summarising and reporting results, and (6) analysing and interpreting results.

### Step 1: Defining the research questions

The primary goal of this review was to identify improvements addressing referral gaps from PHC to SHC. Specifically, we sought to map existing strategies intended to improve the effectiveness, efficiency and overall quality of referrals. We also explored how these strategies align with the Quintuple Aim, focusing on both patient-level and system-level outcomes. Eligibility criteria were guided by a Population–Concept–Context (PCC) framework,^34^ detailed as follows:

- Population: Studies examining referrals originating from primary care physicians (eg, general practitioners, family doctors) to secondary care clinicians (eg, hospital-based SHCs or advanced-level practitioners).
- Concept: Interventions or improvements aimed at addressing referral gaps, whether related to knowledge deficits, knowledge–practice gaps or organisational challenges.
- Context: PHC settings such as general practices and family medicine clinics, where referral decisions typically initiate.

Further details of our inclusion and exclusion criteria are provided in online supplemental file 3.

### Step 2: Identifying relevant studies

A search strategy was developed in collaboration with a medical librarian, with full search strings documented in online supplemental file 2. Searches were conducted in PubMed, Scopus and CINAHL, covering publications from January 2013 to May 2024. We used EndNote to manage citations and remove duplicates automatically. The remaining records were transferred to Rayyan for manual deduplication, yielding a final set of unique records that proceeded to title and abstract screening.

### Step 3: Study selection

Before conducting the main screening, we ran a small pilot in Rayyan to refine our inclusion and exclusion criteria (online supplemental file 3) and ensure reviewer alignment. The unique records were then randomly divided into two groups of approximately equal size. In each group, two reviewers independently assessed each citation—classifying articles as ‘include’, ‘exclude’ or ‘maybe’ and recording rationales.

Discrepancies were resolved through discussion within and between reviewer pairs. Full-text articles were retrieved and assessed for eligibility using the same criteria, with unclear cases resolved through consultation with coauthors. Full-text screening was performed by the first author (KOB); cases where eligibility was unclear were resolved through consultation with coauthors.

### Step 4: Charting data

A standardised charting form was used to systematically capture information from each included study,^36–120^ reflecting the PCC framework. This form included basic publication details (eg, author, year, design), the referral context (eg, country, health domain), the targeted step(s) of the referral process, the rationale for the intervention, specific referral problems addressed and all reported outcomes. We also recorded any mention of patient experience, costs, health equity or provider well-being. The completed extraction table is available in online supplemental file 5.

Data extraction was partially automated using Elicit, an artificial intelligence (AI)-based platform that parsed each full text and populated a spreadsheet.^121^ All Elicit-extracted data were subsequently verified systematically against the source articles by the first author (KOB); only minor discrepancies were identified, and these were resolved by prioritising the source article content. While systematic verification was applied to all included studies, the potential for confirmation bias—arising from reading the AI-generated extraction prior to verification—cannot be fully excluded.

### Step 5: Collating, summarising and reporting results

After data extraction, we organised our findings using two complementary frameworks to highlight both the nature of referral pathway gaps and the improvements proposed. Domain alignment with the Quintuple Aim was determined by assessing whether each included study reported outcomes or described aspects relevant to each of the five domains, using structured analytical columns applied within Elicit. Improvement strategies were inductively grouped into eight broad categories reflecting common intervention types across the included studies. Both the domain mapping and categorisation were conducted by the first author (KOB) with continuous input from coauthors throughout the process. The two frameworks were as follows:

1. Standard referral pathway versus Quintuple Aim (table 1): We first compared each stage of the referral pathway—patient assessment and decision to refer, information transfer to SHC, coordination and tracking, specialty access and appointment, and feedback/close-the-loop—against the five domains of the Quintuple Aim (patient experience, population health, costs, provider well-being and health equity). This approach provided a structured view of how deficits at each stage can adversely affect multiple dimensions of care.
2. Referral improvements versus Quintuple Aim (table 2): We then grouped interventions into eight broad improvement categories (eg, educational interventions, electronic referral systems) and again used the Quintuple Aim domains to assess their reported impact and any trade-offs. By linking each improvement category to relevant referral gaps, we could evaluate whether interventions effectively addressed core problems and whether they inadvertently created new challenges in other domains (eg, high startup costs or workflow burden).

**Table 1:** Comparison of standard referral pathway gaps and their impact on the Quintuple Aim

Referral step	Standard referral pathway	Gaps in current practice	Impact on health outcomes	Impact on patient experience	Impact on costs	Impact on provider well-being	Impact on health equity
Patient assessment and decision to refer	Timely recognition of referral needs. Adequate use of guidelines.	Late or defensive referrals. Inconsistent guideline use. Limited PCP awareness or training.	Higher rates of complications if referrals delayed. Possible advanced disease on presentation.	Longer wait times if urgent needs go unrecognised. Anxiety/confusion for patients uncertain about care pathway.	Extra costs from unnecessary referrals or tests. Potential burden on specialised services.	PCP frustration from uncertainty. Fear of missing diagnoses.	Rural/underserved patients face greater delays. Inconsistent guideline use exacerbates care disparities.
Information transfer to SHC	Clear reason for referral. Complete referral details (history, labs, imaging).	Poor-quality referral letters. Incomplete or unclear clinical information. Non-standardised templates.	Incomplete info leads to delayed or inaccurate diagnosis.	Repeated questioning. Frustration if patients must retell history.	Additional administrative overhead. Potential retests or follow-up appointments.	SHCs receive partial data, must spend extra time clarifying. PCPs feel burdened by complex referral requirements.	Communication gaps can disproportionately affect patients with language or literacy barriers.
Coordination and tracking of referral	Clear follow-up responsibilities. Reliable tracking and scheduling.	Lost or untracked referrals. Fragmented IT systems. No mechanism to track attendance or outcomes.	Missed diagnoses if referrals go unmonitored. Treatment delays increase morbidity.	Patients sense disjointed care. Confusion on next steps or who is accountable.	Wasted resources from repeat appointments or tests. Inefficient use of SHCs.	Administrative overload. Staff demotivation if the process is unmanageable.	Low-literacy or remote populations at higher risk for missed follow-up.
Specialty access and appointment	Timely SHC access. Urgent cases prioritised, others scheduled promptly.	Long wait times. Geographical barriers. Inadequate pathways and infrastructures.	Delayed diagnoses and disease progression. Higher complication rates.	Anxiety and dissatisfaction from long waits. Difficulty travelling or coordinating appointments.	Higher healthcare spending on late. Stage interventions. Overburdened SHC clinics.	Emotional strain on providers seeing advanced disease. Frustration with limited referral resources.	Disadvantaged populations more likely to experience appointment delays and related complications.
Feedback/close-the-loop	Clear SHC feedback to PCP. Integrated care plan and follow-up.	Limited or delayed communication back to PCP. Incomplete or missing SHC notes. Failure to close referral loop.	Gaps in follow-up hinder continuity of care. Potential for missed labs or unaddressed findings.	Patients remain unsure about next steps. Repeated visits or contradictory advice.	Extra costs from duplicate visits/tests. No accountability for unnecessary referrals.	PCPs lack input to improve future referrals. SHC overloaded with back-and-forth clarifications.	Vulnerable patients at higher risk for incomplete follow-up. Inequity risk without structured feedback.

This table presents a structured overview of the referral process by contrasting the standard referral pathway (first two columns) with the current gaps and their impact (remaining columns). The challenges are analysed through the Quintuple Aim perspective.

IT, information technology; PCP, primary care physician; SHC, secondary healthcare.

**Table 2:** Referral improvement categories, examples and their main benefits and trade-offs across the Quintuple Aim

Improvement category	Studies (n)	Intervention types	Main benefits (domain)	Main trade-offs (domain)
(1) Educational and training interventions	18	Teaching/training sessions; interactive case-based workshops; peer-to-peer coaching	Earlier detection (population health); better-informed decisions (patient experience)	Training workload (provider well-being); equity rarely assessed (health equity)
(2) Electronic referral systems	26	E-referral platforms; referral manager/EHR modules; computerised booking systems	Fewer lost referrals (costs); faster access and tracking (patient experience)	High setup cost (costs); digital divide (health equity)
(3) Structured referral templates	12	Structured templates and proformas; referral-writing software; checklists	Better diagnoses (population health); clearer communication (patient experience)	Rigid for complex cases (population health); provider well-being rarely assessed (provider well-being)
(4) Clinical decision support systems	8	Decision support in EHR; risk-based triage tools; guideline prompts	More appropriate referrals (population health); fewer unnecessary referrals (costs)	Alert fatigue (provider well-being); equity rarely assessed (health equity)
(5) Referral management centres	3	Central triage/substitution services; telephone-based referral management	Streamlined workflows (costs); fewer unnecessary visits (patient experience)	Can feel restrictive (patient experience); uneven integration (health equity)
(6) Process improvements and protocols	18	Guideline implementation; new or updated referral pathways; local process changes	Timely, targeted referrals (population health); fewer unnecessary tests (costs)	Administrative burden (provider well-being); little regional adaptation (health equity)
(7) Feedback and quality assurance mechanisms	5	Written feedback and audits; performance dashboards; triage feedback	Higher referral quality (population health); fewer inappropriate referrals (costs)	Provider well-being not reported (provider well-being); effects varied by delivery and engagement
(8) Resource allocation and workforce expansion	6	Additional staff or roles; telemedicine and eConsult expansion	Serves underserved areas (health equity); better access (patient experience)	Hiring/training cost (costs); hard to sustain (provider well-being)

Domains are the five Quintuple Aim aims: patient experience, population health, costs, provider well-being, health equity. Entries reflect observations reported across included studies, not appraised effectiveness.

EHR, electronic health record.

These two analytical layers—mapping referral pathway steps and mapping improvements—allowed us to integrate diverse findings, underscore common patterns and reveal underexplored areas.

A plain language summary is available as online supplemental file 6.

### Patient and public involvement

None.

## Results

The searches retrieved 1743 records in total (PubMed: n=641; Scopus: n=779; CINAHL: n=323). After automated deduplication using EndNote (n=669 duplicates removed) and manual deduplication in Rayyan (n=175 additional duplicates), 899 unique records proceeded to title and abstract screening. In the first screening group, 120 conflicts led to 57 articles advancing to full-text review; in the second group, 112 conflicts yielded 97 inclusions. Overall, 154 records advanced to full-text review, of which 148 were successfully retrieved. Applying the eligibility criteria, 85 studies were included for data extraction. The selection process is illustrated in the PRISMA flow diagram (online supplemental file 4).

The overall characteristics of the 85 included studies are first summarised in table 3. These studies span diverse designs (observational research, randomised controlled trials, quality improvement initiatives and qualitative analyses) and vary widely in geography (primarily high-income countries such as the USA, the UK and Canada). Sample sizes range from small pilot evaluations involving a few dozen referrals to large datasets exceeding 450 000 referrals. Clinical areas also differ substantially; while musculoskeletal and dermatology referrals feature prominently, specialties such as oncology, cardiology and endocrinology are also represented. Few studies focus on multimorbidity or use a broader PHC perspective.

**Table 3:** Overall characteristics of the 85 included studies

Characteristic	Findings/categories
Study designs	Observational (n=38)
	RCT (n=10)
	Quality Improvement (n=12)
	Pilot (n=8)
	Quasi-experimental (n=3)
	Descriptive (n=4)
	Time series (n=3)
	Qualitative (n=4)
	Other (n=3)
Geographical distribution	Europe (n=41; mostly UK, Norway, Netherlands)
	North America (n=29; USA and Canada)
	Oceania (n=6; Australia and New Zealand)
	Africa (n=3; Ghana, Nigeria, Egypt)
	Asia (n=3; Hong Kong, Saudi Arabia, Qatar)
	South America (n=3; Brazil)
Number of referrals	Range: 12–457 164
	Median: 420.5 (IQR: 156–887); range: 12–457 164; Mean: ~8797 (SD: ~60 000; highly skewed distribution); 70 studies specify totals; 8 do not specify; seven report referral rates only
	Median: 420.5
	SD: ~60 000
	70 studies specify totals; 8 do not specify; 7 report referral rates only
Study duration	76 studies report length
	Range: 60–4015 days (~11 years)
	Mean: 618 days
	Median: 530 days
	SD: ~1000 days
Health domains	Musculoskeletal (n=18)
	Dermatological (n=17)
	Cardiovascular (n=15)
	Oncological (n=10)
	Neurological/psychological (n=9)
	Renal (n=7)
	Endocrine (n=4)
	Respiratory (n=3)
	Ophthalmological (n=3)
	Infectious diseases (n=3)
	Obstetrics/gynaecology (n=4)
	Other (n=13)
	Not specified (n=6)
Common outcomes assessed	Referral volume and rates (eg, 28 report volume; 48 report rates)
	Referral quality and appropriateness (eg, 9 measure letter quality; five track % inappropriate)
	Timeliness and efficiency (11 report wait times; 11 measure follow-up)
	Cost and economic impact (cost savings n=5; other economic metrics n=3–4)
	Clinical and diagnostic outcomes (eg, cancer detection n=8; diagnostic accuracy n=5)
	Patient and provider satisfaction/behaviour (various measures in 2–4 studies each)

RCT, randomised controlled trial.

**Figure 1:**
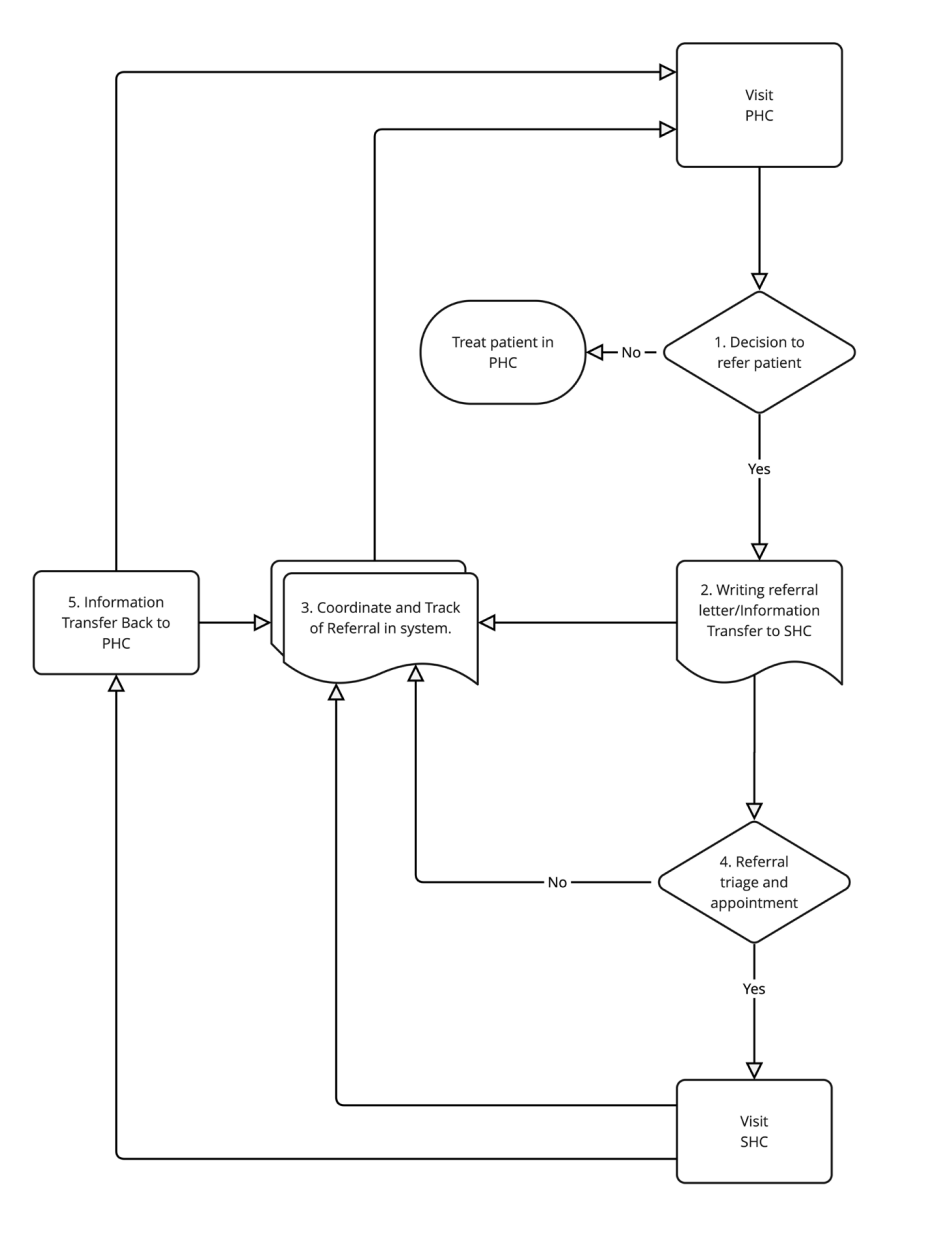
Standard primary-to-secondary care referral pathway. The figure illustrates five sequential steps of a typical PHC-to-SHC referral: (1) patient assessment and decision to refer; (2) information transfer to SHC; (3) coordination and tracking; (4) specialty access and appointment; and (5) feedback/close-the-loop. PHC, primary healthcare; SHC, secondary healthcare.

Following this overview, we present our findings in two complementary ways: table 1 depicts the standard referral pathway—illustrated in figure 1—to show how each step intersects with the five domains of the Quintuple Aim. Building on that, table 2 categorises eight improvement types and examines their reported outcomes and trade-offs in relation to the Quintuple Aim.

### Referral pathway gaps and their impact on the Quintuple Aim

As shown in figure 1, a referral pathway typically proceeds through five steps: (1) patient assessment and decision to refer, (2) information transfer to SHC, (3) coordination and tracking, (4) specialty access and appointment, and (5) feedback/close-the-loop. Summarised in table 1, across all five steps, the included studies identified recurring gaps—among them delays, incomplete or unclear documentation, insufficient coordination and limited follow-up mechanisms.

1. Patient assessment and decision to refer: Late referrals or ‘defensive referrals’ (ie, referrals made primarily to avoid missing a potential diagnosis ‘just in case’) commonly stemmed from unclear guidelines, knowledge gaps in primary care or limited support for clinical decision-making.^36 101 112^ Such patterns were linked to downstream effects like advanced disease at presentation (when truly urgent cases were overlooked among non-urgent referrals) and eroded patient experience (eg, anxiety arising from unclear care plans).
2. Information transfer to SHC: Several studies reported referral letters lacking essential clinical data or containing incomplete histories.^57 77^ Incomplete referral information undermined timely diagnosis, contributed to patient frustration (due to repeated questioning) and increased provider dissatisfaction by requiring SHCs to devote extra time clarifying missing details.
3. Coordination and tracking: Fragmented information technology (IT) systems, poor interoperability and uncertain accountability often resulted in lost or unmonitored referrals.^87 98 120^ These lapses compromised patient safety and led to inefficiencies, including redundant testing, wasted appointments and additional workload for administrative staff.
4. Specialty access and appointment: Long wait times—particularly in high-demand specialties—represented a near-universal concern. Geographical barriers and inconsistent triage standards exacerbated inequities, especially for rural or vulnerable populations.^51 72 90^ Delayed access to SHC consultations, in turn, risked disease progression or more extensive treatments later on.
5. Feedback/close-the-loop: After SHC evaluation, the absence of a systematic feedback loop back to primary care hindered continuity. Several studies noted incomplete or delayed communication regarding diagnosis, medication changes or recommended follow-up, thereby perpetuating fragmented care and elevating the risk of missed findings.^96 118^

Collectively, these five categories of gaps resonated across all domains of the Quintuple Aim. Delayed or defensive referrals jeopardised health outcomes, while poor information transfer and long wait times undermined patient experience. Redundant investigations and inefficiencies incurred unnecessary costs, and heavy administrative burdens contributed to provider dissatisfaction. Finally, inadequate coordination and triage criteria widened health equity gaps, disproportionately affecting resource-limited or geographically remote populations.

### Improvements and their alignment with the Quintuple Aim

Below, we briefly describe eight broad categories of referral improvements identified in our scoping review (see table 2). Each category addresses specific referral gaps and demonstrates how interventions may align with (or inadvertently undermine) one or more dimensions of the Quintuple Aim: patient experience, population health, costs, provider well-being and health equity.

1. Educational and training interventions: Several studies employed teaching sessions, interactive workshops or peer coaching to mitigate knowledge gaps and reduce inappropriate or delayed referrals.^36 101 109^ While improved referral timeliness and greater adherence to guidelines were common benefits, sustained impact often required ongoing education. Without continued reinforcement, these gains diminished over time, potentially limiting their long-term effect on both patient care and provider well-being.
2. Electronic referral systems: Electronic referral (e-referral) platforms emerged as a frequently cited strategy to standardise documentation and facilitate scheduling or follow-up.^86 87 119^ Effective implementation generally reduced lost referrals, streamlined administrative tasks and improved patient experience via faster scheduling. Nevertheless, studies highlighted high startup costs, variable user acceptance and limited digital infrastructure in some settings—factors that, if unaddressed, may negatively influence costs, provider satisfaction and health equity.
3. Structured referral templates: Many studies recommended standardised referral templates to ensure essential clinical information was included.^39 46 57^ Templates typically improved referral letter quality, supported more accurate triage and trimmed costs by cutting down on duplicated efforts. At the same time, rigid templates sometimes proved challenging for complex or unusual cases, highlighting the need to balance uniform documentation with individualised patient needs.
4. Clinical decision support systems (CDSS): By integrating automated guidelines, pop-up alerts or risk-based triage tools into electronic health records, CDSSs helped standardise referral decisions.^58 63^ These systems often reduced wait times, improved referral appropriateness and alleviated provider anxiety regarding missed diagnoses. Studies nevertheless cautioned against overreliance on automated prompts, noting risks such as ‘alert fatigue’ and the possibility that unique or atypical cases might be overlooked.
5. Referral management centres: Centralised referral hubs or telephone-based triage lines provided an alternative to direct referral to SHC clinics.^38 42 120^ By screening referrals for urgency and complexity, these centres often saved costs and improved patient satisfaction. However, some populations felt that such coordination limited their freedom to access secondary care, potentially affecting health equity if certain groups found the additional triage steps burdensome.
6. Process improvements and protocols: A range of local initiatives—such as updating guidelines, creating fast-track processes or introducing condition-specific referral pathways—improved timeliness and adherence to best practices.^55 59 65 89 112^ While these measures commonly enhanced health outcomes, consistent uptake required adequate provider training, clear guidelines and ongoing resources to sustain the changes.
7. Feedback and quality assurance mechanisms: Structured feedback loops, encompassing referral audits or direct communication channels between primary and secondary care, proved crucial for continuous improvement.^49 110 120^ Clear SHC feedback reduced referral rejections and built trust across care levels. However, implementing these mechanisms sometimes placed additional burdens on already strained providers, underscoring the importance of adequate support to maintain provider well-being.
8. Resource allocation and workforce expansion: Finally, facing workforce shortages and crowded clinics, some studies described adding staff or extending telemedicine services—particularly in rural or underserved regions—to enhance access and cut wait times.^67 72 90^ While these interventions often produced immediate gains in patient experience and equity, sustaining them over time required consistent funding, ongoing training and supportive organisational policies.

### Synthesis and key observations

Taken together, the findings indicate that no single intervention uniformly addresses the broad spectrum of referral challenges. Rather, multifaceted strategies—combining standardised templates, educational programmes, robust IT infrastructures and active feedback loops—were more frequently associated with reported improvements across multiple Quintuple Aim domains. Although gains in costs and population health outcomes were more commonly reported, gaps remain, especially regarding equitable access for underserved populations and mitigating the administrative burden on frontline providers.

## Discussion

### Key findings and their implications

Mapping the referral pathway gaps (table 1) and specific interventions (table 2) to the Quintuple Aim shows that effective referral optimisation requires more than just technical fixes—organisational readiness, comprehensive provider training and continuous quality assurance also play crucial roles. This scoping review identified a broad range of interventions aimed at improving the referral process from PHC to SHC. Key insights include: (1) a dominant emphasis on efficiency, with less focus on provider well-being and health equity; (2) significant gains in referral completeness, timeliness and appropriateness, yet limited clarity around patient outcomes and sustainability; (3) some studies reported increased administrative burdens as an unintended consequence of certain interventions; and (4) limited evidence was identified from low-resource or rural settings, suggesting that identified improvements may not reach all populations equally.

#### Provider well-being and administrative burdens

Although increasing referral efficiency is a frequent objective, few interventions directly measured their impact on provider workload or job satisfaction. Initiatives emphasising electronic systems or standardisation may inadvertently shift administrative tasks to primary care staff, exacerbating burnout in an already pressured workforce.^81 86 122 123^ Furthermore, many referral improvements target individual conditions or specialties, limiting their utility in broader PHC settings where clinicians manage multiple diseases simultaneously. Future studies should consider both qualitative and quantitative evaluations of workflow changes, assessing how new processes influence cognitive load, job satisfaction and overall provider well-being.

#### Health equity considerations

Persistent inequities in referral access emerged across different interventions, often tied to digital readiness or socio-economic factors.^93 124^ Telehealth services, for instance, can extend specialty access in rural settings but may erect barriers for patients lacking internet connectivity or sufficient health literacy.^69 72^ Similarly, language barriers and cultural mismatches in referral documentation disproportionately affect non-native speakers, who may struggle to navigate the system.^113^ Addressing these gaps requires equity-focused design and evaluation—such as culturally adapted communication materials, low-tech alternatives where digital infrastructure is weak and patient navigation programmes to support underserved groups. Only by integrating these measures can referral pathways achieve comprehensive alignment with the Quintuple Aim.

### Placement in the context of existing literature

Past reviews often highlighted strategies that lower referral volumes or expedite SHC triage, paying limited attention to PHC-driven improvements and underemphasising systemic factors like provider well-being or structural inequities.^10 14–16^ Our findings extend this work by explicitly evaluating referral interventions through the Quintuple Aim framework, revealing persistent gaps in equity and limited empirical assessment of provider experience. Moreover, while many interventions thrive in high-resource settings, studies in low-income or rural contexts remain sparse, underscoring the need for adaptable improvements that account for diverse infrastructures and patient populations.^55 93 124^ Even widely adopted approaches—such as e-referrals and standardised templates—can prove insufficient for complex real-world scenarios, especially for patients with multiple comorbidities or atypical presentations.^112 115^

### Limitations

As a scoping review, this study maps referral process improvements but does not assess intervention effectiveness; findings should therefore be interpreted as descriptive rather than conclusive. Full-text screening was performed by a single reviewer (KOB), which, despite consultation with coauthors for unclear cases, may have introduced selection bias. This approach is, however, consistent with methodological guidance for expedited evidence synthesis, which acknowledges single-reviewer screening as an acceptable efficiency measure when supplemented by co-author consultation.^125^ The restriction to English-language publications from 2013 to May 2024 may have excluded relevant evidence from healthcare systems in non-English-speaking countries, and their distinct referral challenges and solutions may consequently be lacking in this scoping review.

Another limitation is the heterogeneity of study designs, which complicates direct comparisons. Referral interventions were evaluated using diverse metrics, including referral appropriateness, timeliness and cost savings, but fewer studies reported patient-reported outcomes or long-term impacts. The use of the Quintuple Aim as an analytical framework meant that outcomes not mapped onto its five predefined domains may have received less attention in the synthesis. Additionally, although Elicit-assisted extraction was systematically verified against source articles, the potential for confirmation bias during verification cannot be fully excluded.^121^ Finally, stakeholder validation (eg, engagement with PCPs, SHCs and patients) was not incorporated, limiting the real-world applicability of findings.

### Future research directions

Based on the gaps identified in this review, we propose the following directions for future research:

1. Longitudinal impact assessments: Future studies should examine the sustainability of referral improvements over time, tracking whether initial efficiency gains persist or lead to unintended consequences (eg, provider fatigue, patient disengagement).
2. Provider well-being research: Given the increasing administrative burden on PCPs, future work should systematically assess how referral interventions influence clinician stress, workload and job satisfaction.^122 123^
3. Equity-focused interventions: More research is needed to evaluate whether referral improvements exacerbate or mitigate healthcare disparities, particularly in rural and underserved populations.^93 124^
4. Patient-centred outcomes: Few studies assess how referral process improvements impact patient experience beyond access speed; future work should incorporate patient-reported measures of satisfaction, trust and health literacy.
5. Cross-sector collaboration: Research should explore interdisciplinary approaches to referral coordination, incorporating perspectives from primary care, secondary care, patient advocacy groups and policymakers.

## Conclusion

This review highlights both the progress and persistent gaps in optimising referral pathways from PHC to SHC. While technological and process-based interventions were more frequently associated with reported improvements in referral efficiency, their long-term sustainability and impact on provider well-being and health equity were less frequently evaluated in the included studies, representing underexplored areas in the literature. Moving forward, referral improvements must align with the full spectrum of the Quintuple Aim, ensuring that efficiency gains do not come at the expense of workforce sustainability, equitable access or patient-centred care.

## Supplementary material

10.1136/bmjopen-2025-105430 online supplemental file 1

10.1136/bmjopen-2025-105430 online supplemental file 2

10.1136/bmjopen-2025-105430 online supplemental file 3

10.1136/bmjopen-2025-105430 online supplemental file 4

10.1136/bmjopen-2025-105430 online supplemental file 5

10.1136/bmjopen-2025-105430 online supplemental file 6

## Data Availability

All data relevant to the study are included in the article or uploaded as supplementary information.
