# Supplementary material for: Mapping the literature on improving primary-to-secondary care referrals through the lens of the Quintuple Aim framework for healthcare improvement: a scoping review
Source: BMJ Open. 2026 Aug 3;16(8):e105430. doi: 10.1136/bmjopen-2025-105430 (PMC13435927; doi:10.1136/bmjopen-2025-105430)
Supplement: Supplementary file 1 [file bmjopen-2025-105430.reviewer_comments.pdf]

PEER REVIEW HISTORY

BMJ Open publishes all reviews undertaken for accepted manuscripts. Reviewers are asked to complete a checklist review form and are provided with free text boxes to elaborate on their assessment. These free text comments are reproduced below.

ARTICLE DETAILS

Title (Provisional)

Mapping the literature on improving primary-to-secondary care referrals through the lens of the Quintuple Aim framework for healthcare improvement: a scoping review

Authors

Björkman, Karl Oskar; Rejler, Martin; Masterson, Daniel; Arvidsson, Eva; Fristedt, Sofi

---

VERSION 1 - REVIEW

**Reviewer**	**1**
**Name**	**Mamo, Nick**
**Affiliation**	**University Medical Centre Groningen**
**Date**	**02-Apr-2026**
**COI**	**None**

Was only one reviewer involved in the full text screening? If so, this seems like a limitation/risk of bias that should be addressed (though I appreciate this is a scoping review not a systematic review).

Language/grammar/punctuation issues:

Strengths and limitations point 6 - review English. Suggestion: AI was utilized to assist with parts of the data extraction process. While there was subsequent manual verification, the potential for AI-specific biases in study interpretation or charting cannot be entirely ruled out.

**Reviewer**	**2**
**Name**	**Beischer, Susanne**
**Affiliation**	**Sahlgrenska Academy**
**Date**	**25-May-2026**
**COI**	**None**

Summary of the manuscript and overall assessment

I would like to thank the authors for the opportunity to review this manuscript. The manuscript addresses an important aspect of care coordination between primary and secondary healthcare. This is a relevant and ambitious scoping review using the Quintuple Aim framework.

Overall, the manuscript follows a logical structure, and the different sections are generally well aligned. However, some aspects of the manuscript could be clarified to strengthen the overall coherence of the manuscript.

Major comments

1. The PCC framework and analytical approach clearly include both referral pathway gaps and referral improvement strategies. However, the stated aim and research questions primarily emphasize referral improvements. Given the substantial role of the gap analysis throughout the results (Table 1), consider whether the mapping of key referral pathway gaps should also be more explicitly reflected in the introduction and/or stated aim.

2. In the Methods section ("Step 5: Collating, Summarizing, and Reporting Results"; page 9–11), please consider further clarifying the analytical process used to map referral gaps and improvement strategies to the Quintuple Aim domains (e.g., categorization approach, reviewer involvement, and how alignment with domains was determined).

Minor comments

Abstract

- Please review the aim in relation to the comments provided below.

- Consider providing slightly more methodological detail in the abstract regarding the PCC framework, eligibility criteria, and how referral gaps and improvements were mapped and categorized according to the Quintuple Aim.

Introduction

- Page (P) 6, Line (L) 52-5. The reference to the review protocol may disrupt the flow, as the study aim has not yet been clearly established. It may be clearer to first present the rationale and aim, and then refer to the protocol and registry search.

The paragraph may benefit from improved structure. Consider starting with the overview of existing systematic reviews and concluding with a clear statement of the knowledge gap. For example, the statement regarding the absence of protocols could be repositioned to the end of the paragraph to strengthen the narrative flow.

Methods

- P8, L:6-15: The reporting of the search results and deduplication process is clear and transparent. However, according to PRISMA-ScR guidance (e.g., Item 7 and 9), it may be preferable to separate the description of the search process from the reporting of numerical results.

Specifically, the Methods section could focus on the search strategy and procedures, while detailed record counts may be more appropriately presented in the Results section and PRISMA flow diagram.

Revising this structure could improve alignment with PRISMA-ScR reporting standards and enhance readability by clearly distinguishing between methodology and study findings.

Consider also moving the sentence "A PRISMA flowchart illustrates this process (Supplementary file 4)" to the Results section

- P8, L17-33. A similar issue appears here, where detailed numerical results (e.g., number of conflicts and included records) are reported. Consider moving these results to the Results section, while keeping the Methods focused on describing the screening procedure.

- P8, L47-50: The use of AI-assisted data extraction (Elicit) is interesting and relevant. However, it would be helpful to clarify how ambiguities were identified in practice. For example, were all extracted data verified against the original articles, or only selected cases? Providing a brief example or additional detail on how discrepancies or uncertainties were detected and resolved would improve transparency and reproducibility.

- P9, L 49: "figure 1" should be capitalized as "Figure 1" for consistency.

Results

- P11, L52-60: The section "Synthesis and Key Observations" provides a useful overall summary of the findings. However, some statements appear somewhat interpretive and discussion-oriented (e.g., regarding which interventions "yield the strongest gains" or "show promise"). Consider clarifying the distinction between result synthesis and discussion/interpretation to improve structural consistency.

Tables and figures

Figure 1: Please ensure that all abbreviations used in the figure are spelled out below the figure. Please also add a period after each number (e.g., "1. Decision to refer patient").

The figure has low image resolution. Please consider replacing it with a higher-resolution version to improve readability.

Table 1 and 2:

- The tables are comprehensive and well structured; however, they are currently very text-dense. Consider shortening the text within cells and simplifying the formatting to improve readability.
- In addition, please ensure that words are not split across lines or cut off within cells, as this makes the table difficult to read.
- Please ensure consistent terminology, capitalization, and heading style throughout the table (e.g., sentence case for headings).
- Please define all abbreviations used in the tables below the tables.
- Please review the tables for minor language inconsistencies, formatting issues, and heading clarity to improve overall readability.
- Please carefully review Table 2 for formatting inconsistencies and unresolved placeholders. Several entries appear to contain temporary annotations or export artefacts that should be corrected prior to publication (e.g., “96??”, “118??”, and “Emmett, 2022 #181”).

Table 3

Given the highly skewed distribution in the number of referrals (range 12–457,164), consider whether the mean and standard deviation are informative summary measures in this context. The median and range may better reflect the distribution of study sizes.

Supplementary File 5

Although presented as supplementary material, the extraction table is currently very extensive and difficult to navigate. Consider streamlining the content to improve readability and usability for readers, focusing primarily on information relevant to the review objectives and synthesis. Several columns appear overly detailed or repetitive (e.g., bibliographic/exported reference information and extensive abstract-related content), and some information could potentially be condensed or omitted. In addition, the formatting appears inconsistent in places (e.g., varying fonts/styles across columns), and some headings appear database-generated rather than manuscript-oriented (e.g., “Publication Title”).

Discussion

- P12, L 16-24: In the “Key Findings and Their Implications” section, points 3 and 4 appear to describe potential consequences, risks, or broader system implications rather than referral improvements or intervention outcomes per se. Consider revising or

reframing these points to better align with the preceding summary of referral interventions and reported outcomes.

- P13, L 15-32: Some parts of the limitations section could be more specifically linked to the findings and methodological decisions of the present review.

Please remove the bold formatting in the limitations section for consistency with the overall manuscript style.

- P13, L38-52: This section would benefit from more consistent use of referencesConsider using references more consistently throughout this section, or alternatively presenting the points more clearly as the authors' own recommendations.

Conclusion

The conclusion is generally well aligned with the review findings and conceptual framework. However, the statement that the "impact on provider well-being and health equity remain unclear" may benefit from slightly more precise wording, as the review appears to demonstrate that these domains were less frequently evaluated or remain underexplored in the literature, rather than simply unclear. Consider revising the wording to better reflect the descriptive nature of the scoping review findings.

Please remove the bold formatting in the conclusion section for consistency with the overall manuscript style.

Reference list

- Please carefully review the reference list for formatting inconsistencies, export artefacts, and incomplete citation information. Several entries appear to contain parsing or encoding issues in author formatting (e.g., "M, C.; Ic, C.; S, S.", "Ak; e, Y.", and "orf, L."), which should be corrected prior to publication.

- Reference 20 appears to refer to the review protocol; however, it is currently unclear whether this protocol is published, registered, publicly available, or unpublished. Please clarify the status and accessibility of the protocol and provide complete reference information if applicable.

VERSION 1 - AUTHOR RESPONSE

Reviewer 1 (Dr. Nick Mamo, University Medical Centre Groningen)

We thank Reviewer 1 for their careful reading and constructive suggestions.

R1.1 [Reviewer 1]

""Was only one reviewer involved in the full text screening?""

(Addressed with R1.2). Methods Step 3: sentence added clarifying full-text screening performed by KOB; unclear cases resolved through co-author consultation. Tracked change in working manuscript.

R1.2 [Reviewer 1]

> *"“If so, this seems like a limitation/risk of bias that should be addressed (though I appreciate this is a scoping review not a systematic review).”"*

See R1.1. Limitation also addressed in Discussion/Limitations (Phase 7).

R1.3 [Reviewer 1]

> *"“Strengths and limitations point 6 - review English.”"*

(Addressed together with R1.4 and E11). AI sentence (bullet 6) revised.

R1.4 [Reviewer 1]

> *"“Suggestion: AI was utilized to assist with parts of the data extraction process. While there was subsequent manual verification, the potential for AI-specific biases in study interpretation or charting cannot be entirely ruled out.”"*

Bullet 6 revised to: “AI was utilized to assist with parts of the data extraction process; while there was subsequent manual verification, the potential for AI-specific biases in study interpretation or charting cannot be entirely ruled out.” Tracked change in working manuscript.

Reviewer 2 (Dr. Susanne Beischer, Sahlgrenska Academy)

We thank Reviewer 2 for the detailed and methodologically rigorous feedback.

R2.1 [Reviewer 2]

> *"“The PCC framework and analytical approach clearly include both referral pathway gaps and referral improvement strategies.”"*

(Addressed together with R2.2 and R2.3). Aim revised to: “...to explore and map the literature on referral pathway gaps and improvement strategies in PHC-to-SHC referrals, and to categorize both gaps and improvements by applying the Quintuple Aim framework for healthcare improvement.” Tracked changes in working manuscript.

R2.2 [Reviewer 2]

> *"“However, the stated aim and research questions primarily emphasize referral improvements.”"*

(Addressed together with R2.1 and R2.3). See R2.1.

R2.3 [Reviewer 2]

> *"“Given the substantial role of the gap analysis throughout the results (Table 1), consider whether the mapping of key referral pathway gaps should also be more explicitly reflected in the introduction and/or stated aim.”"*

(Addressed together with R2.1 and R2.2). RQ1 revised to: “What referral pathway gaps have been identified in PHC-to-SHC referrals, and which improvements have been studied to address them?” Rationale sentence (para before objective) also revised to include gaps explicitly. Tracked changes in working manuscript.

R2.4 [Reviewer 2]

"“In the Methods section (‘Step 5: Collating, Summarizing, and Reporting Results’; page 9–11), please consider further clarifying the analytical process used to map referral gaps and improvement strategies to the Quintuple Aim domains.”"

(Addressed together with R2.5). Step 5: added sentence explaining domain alignment determined via Elicit structured columns, improvement categories derived inductively, and mapping conducted by KOB with co-author input. Tracked change in working manuscript.

R2.6 [Reviewer 2]

"“Please review the aim in relation to the comments provided below.”"

Abstract Objectives section updated to reflect revised aim (gaps + improvements).

R2.7 [Reviewer 2]

"“Consider providing slightly more methodological detail in the abstract regarding the PCC framework, eligibility criteria, and how referral gaps and improvements were mapped and categorized according to the Quintuple Aim.”"

PCC framework, eligibility criteria, and Quintuple Aim mapping process described in new abstract sections.

R2.8 [Reviewer 2]

"“P 6, L 52-5. The reference to the review protocol may disrupt the flow, as the study aim has not yet been clearly established.”"

(Addressed together with R2.9–R2.12). Protocol sentence moved from start of paragraph to end. See R2.12.

R2.12 [Reviewer 2]

"“For example, the statement regarding the absence of protocols could be repositioned to the end of the paragraph to strengthen the narrative flow.”"

Sentence “During the development of this review’s protocol (20)...” deleted from start of paragraph and reinserted at end of para 62 (after knowledge gap statement), so order is now: existing reviews → gap → need → protocol info. Tracked changes in working manuscript.

R2.13 [Reviewer 2]

"“P8, L:6-15: The reporting of the search results and deduplication process is clear and transparent.”"

Thank you, this comment has been noted.

R2.14 [Reviewer 2]

"“However, according to PRISMA-ScR guidance (e.g., Item 7 and 9), it may be preferable to separate the description of the search process from the reporting of numerical results.”"

(Addressed together with R2.15–R2.19). Record counts removed from Methods Steps 2 and 3 (tracked deletion). New Results opening paragraph with all counts inserted (tracked insertion). PRISMA sentence moved from Methods to Results paragraph. PLS citation corrected to end of Step 5. Tracked changes throughout.

R2.17 [Reviewer 2]

"“Consider also moving the sentence ‘A PRISMA flowchart illustrates this process (Supplementary file 4)’ to the Results section.”"

PRISMA flowchart sentence deleted from Methods; counts and PRISMA reference now in new Results opening paragraph.

R2.20 [Reviewer 2]

> *"“P8, L47-50: The use of AI-assisted data extraction (Elicit) is interesting and relevant.”"*

with R2.21–R2.23). Step 4: expanded to state all Elicit data systematically verified against source articles; minor discrepancies resolved by prioritising source article; potential confirmation bias acknowledged. Tracked change.

R2.24 [Reviewer 2]

> *"“P9, L 49: ‘figure 1’ should be capitalized as ‘Figure 1’ for consistency.”"*

“figure 1” capitalised to “Figure 1” and missing comma added. Tracked change in working manuscript.

R2.25 [Reviewer 2]

> *"“P11, L52-60: The section ‘Synthesis and Key Observations’ provides a useful overall summary of the findings.”"*

(Addressed together with R2.26–R2.27). Interpretive language toned down in Synthesis and Key Observations: “yield the strongest gains” → “were more frequently associated with reported improvements across multiple Quintuple Aim domains”; “show promise” → “more commonly reported”; “underscore” → “indicate”. Tracked changes in working manuscript.

R2.28 [Reviewer 2]

> *"“Figure 1: Please ensure that all abbreviations used in the figure are spelled out below the figure.”"*

Abbreviation note added below Figure 1: “Abbreviations: PHC = primary healthcare; SHC = secondary healthcare.” Tracked insertion in working manuscript.

R2.29 [Reviewer 2]

> *"“Please also add a period after each number (e.g., ‘1. Decision to refer patient’).”"*

Figure 1 has been updated to include a period after each step number (e.g., '1. Decision to refer patient.'). A revised figure with periods is included in the submission files.

R2.30 [Reviewer 2]

> *"“The figure has low image resolution.”"*

Embedded figure replaced in working manuscript with new images in vector and jpeg formats.

R2.31 [Reviewer 2]

> *"“Please consider replacing it with a higher-resolution version to improve readability.”"*

See R2.30.

R2.32 [Reviewer 2]

> *"“Table 1 and 2: The tables are comprehensive and well structured; however, they are currently very text-dense.”"*

Table 1 and 2 cells shortened: 6 cells in Table 1 and 8 cells in Table 2 condensed with tracked changes. All shortened versions preserve the key content while reducing word count.

R2.34 [Reviewer 2]

"“In addition, please ensure that words are not split across lines or cut off within cells, as this makes the table difficult to read.”"

The tables have been reviewed for word-splitting. Remaining layout artefacts are dependent on rendering environment and column widths; these can be resolved at the journal's typesetting stage.

R2.35 [Reviewer 2]

"“Please ensure consistent terminology, capitalization, and heading style throughout the table (e.g., sentence case for headings).”"

Terminology has been standardised throughout (Quintuple Aim, PHC, SHC) and table headings have been reviewed for sentence-case consistency.

R2.36 [Reviewer 2]

"“Please define all abbreviations used in the tables below the tables.”"

Abbreviation notes added below Tables 1 and 2 as tracked insertions (italic, smaller font). Working manuscript updated.

R2.38 [Reviewer 2]

"“Please carefully review Table 2 for formatting inconsistencies and unresolved placeholders.”"

"96??" confirmed as reference 96 (Prichard et al., 2013) and "118??" confirmed as reference 118 (Wahlberg et al.) by cross-checking the reference list. Both corrected with tracked changes in working manuscript.

R2.39 [Reviewer 2]

"“Several entries appear to contain temporary annotations or export artefacts that should be corrected prior to publication (e.g., ‘96??’, ‘118??’, and ‘Emmett, 2022 #181’).”"

All three annotation artefacts have been identified and corrected. '96??' corresponds to reference 96 (Prichard et al., 2013) and '118??' corresponds to reference 118 (Wahlberg et al.), confirmed by cross-referencing the reference list; both have been corrected with tracked changes in the working manuscript. 'Emmett, 2022 #181' is an unformatted EndNote citation corresponding to reference 55 (Emmett et al., Lancet Glob Health, 2022); this citation has been corrected to (55, 64, 88) in ascending numerical order, with a tracked change in the working manuscript.

R2.40 [Reviewer 2]

"“Table 3: Given the highly skewed distribution in the number of referrals (range 12–457,164), consider whether the mean and standard deviation are informative summary measures in this context.”"

(Addressed together with R2.41). Table 3 “Number of Referrals” reordered: Median 420.5 (IQR 156–887, range 12–457,164) now listed first; Mean retained as secondary measure with SD and skewness note. Tracked change in working manuscript.

R2.42 [Reviewer 2]

"“Supplementary File 5: Although presented as supplementary material, the extraction table is currently very extensive and difficult to navigate.”"

(Addressed together with R2.43–R2.45). Supplementary file 5 revised: 10 redundant database-export columns removed (ISSN, DOI, URL, Abstract, Date, Pages, Issue, Volume, Num Pages, Publication Title); 22 columns retained with manuscript-oriented headings; Quintuple Aim columns renamed as QA domain 1–5; typo in column heading fixed. Saved as “Supplementary file 5 - Extraction table revised.xlsx” in 05_Supplementary_files_revised/.

R2.46 [Reviewer 2]

> *"“P12, L 16-24: In the ‘Key Findings and Their Implications’ section, points 3 and 4 appear to describe potential consequences, risks, or broader system implications rather than referral improvements or intervention outcomes per se.”"*

(Addressed together with R2.47). Discussion point (3) revised: “some studies reported increased administrative burdens as an unintended consequence of certain interventions”. Point (4) revised: “limited evidence from low-resource or rural settings, suggesting identified improvements may not reach all populations equally.” Tracked changes in working manuscript.

R2.48 [Reviewer 2]

> *"“P13, L 15-32: Some parts of the limitations section could be more specifically linked to the findings and methodological decisions of the present review.”"*

Para 128 revised: specificity added for single-reviewer screening and English-language restriction. Para 129: Stark style removed; AI sentence already revised in Phase 4. Tracked changes in working manuscript.

R2.49 [Reviewer 2]

> *"“Please remove the bold formatting in the limitations section for consistency with the overall manuscript style.”"*

“Stark” character style (bold) removed from para 129 runs (“heterogeneity of study designs”, “patient-reported outcomes”, “long-term impacts”, “stakeholder validation”) using tracked rPrChange. Working manuscript updated.

R2.50 [Reviewer 2]

> *"“P13, L38-52: This section would benefit from more consistent use of references.”"*

(Addressed together with R2.51). Framing sentence inserted before the Future Research Directions list: “Based on the gaps identified in this review, we propose the following directions for future research:” Tracked insertion in working manuscript.

R2.51 [Reviewer 2]

> *"“Consider using references more consistently throughout this section, or alternatively presenting the points more clearly as the authors’ own recommendations.”"*

See R2.50. Bold (Stark style) also removed from Future Research headings.

R2.52 [Reviewer 2]

> *"“The conclusion is generally well aligned with the review findings and conceptual framework.”"*

Thank you, this comment has been noted and addressed in the revised manuscript.

R2.53 [Reviewer 2]

> *"“However, the statement that the ‘impact on provider well-being and health equity remain unclear’ may benefit from slightly more precise wording.”"*

(Addressed together with R2.54–R2.55). “show promise in improving efficiency” revised to “were more frequently associated with reported improvements in referral efficiency”. “remain unclear” revised to “were less frequently evaluated in the included studies, representing underexplored areas in the literature”. Tracked changes in working manuscript.

R2.56 [Reviewer 2]

"“Please remove the bold formatting in the conclusion section for consistency with the overall manuscript style.”"

“Stark” character style (bold) removed from para 137 runs using tracked change. Working manuscript updated.

R2.57 [Reviewer 2]

"“Please carefully review the reference list for formatting inconsistencies, export artefacts, and incomplete citation information.”"

Reference 28: Aubin M et al. corrected. Reference 79: Chng M, Coulter IC, Surash S — all three authors confirmed from the published PDF and corrected with tracked changes.

R2.58 [Reviewer 2]

"“Several entries appear to contain parsing or encoding issues in author formatting (e.g., ‘M, C.; Ic, C.; S, S.’, ‘Ak; e, Y.’, and ‘orf, L.’), which should be corrected prior to publication.”"

See R2.57. All author names now verified and corrected.

R2.59 [Reviewer 2]

"“Reference 20 appears to refer to the review protocol; however, it is currently unclear whether this protocol is published, registered, publicly available, or unpublished.”"

(Addressed together with R2.60). Reference 20 updated from unpublished work to: “Björkman KOB, Fristedt S, Masterson D, Rejler M, Arvidsson E. The improvements of the referral system: a scoping review protocol. Open Science Framework. 2024. Available from: https://osf.io/87htv/” Tracked change in working manuscript.

R2.60 [Reviewer 2]

"“Please clarify the status and accessibility of the protocol and provide complete reference information if applicable.”"

See R2.59. Protocol confirmed as registered March 2024 (OSF file timestamp). Status: publicly accessible pre-registration.

VERSION 2 - REVIEW

**Reviewer**	**1**
**Name**	**Mamo, Nick**
**Affiliation**	**University Medical Centre Groningen**
**Date**	**07-Jul-2026**
**COI**	

Thank you for the detailed responses and addressing of comments.
