# Supplementary material for: Mapping the literature on improving primary-to-secondary care referrals through the lens of the Quintuple Aim framework for healthcare improvement: a scoping review
Source: BMJ Open. 2026 Aug 3;16(8):e105430. doi: 10.1136/bmjopen-2025-105430 (PMC13435927; doi:10.1136/bmjopen-2025-105430)
Supplement: Supplementary file 3 [file bmjopen-16-8-s002.docx]

# Search Queries

## Cinahl

| Search number | Query | Results |
| --- | --- | --- |
| 1 | TI Referral* | 11,070 |
| 2 | AB Improve*" OR AB Intervent* OR AB Innovat* | 1,085,404 |
| 3 | AB "General Practice*" OR AB "Family Practice*" OR AB "Primary Care*" OR AB "Family Medicine*" OR AB "Primary Health Care*" | 89,555 |
| 4 | ( (1 AND 2 AND 3) ) | 425 |
| 5 | ( (1 AND 2 AND 2) ) AND PY 2013-2024 | 323 |

## Scopus

| Search number | Query | Results |
| --- | --- | --- |
| 1 | TITLE ( referral* ) | 27,447 |
| 2 | ( TITLE-ABS ( improve* ) OR TITLE-ABS ( intervent* ) OR TITLE-ABS ( innovat* ) ) | 12,469,522 |
| 3 | ( TITLE-ABS ( "General Practice" ) OR TITLE-ABS ( "Family Practice" ) OR TITLE-ABS ( "Primary Care" ) OR TITLE-ABS ( "Family Medicine" ) OR TITLE-ABS ( "Primary Health care" ) ) | 271,947 |
| 4 | TITLE ( referral* ) AND ( TITLE-ABS ( improve* ) OR TITLE-ABS ( intervent* ) OR TITLE-ABS ( innovat* ) ) AND ( TITLE-ABS ( "General Practice" ) OR TITLE-ABS ( "Family Practice" ) OR TITLE-ABS ( "Primary Care" ) OR TITLE-ABS ( "Family Medicine" ) OR TITLE-ABS ( "Primary Health care" ) ) | 1,192 |
| 5 | TITLE ( referral* )  AND ( TITLE-ABS ( improve* ) OR TITLE-ABS ( intervent* ) OR TITLE-ABS ( innovat* ) )  AND ( TITLE-ABS ( "General Practice" ) OR TITLE-ABS ( "Family Practice" ) OR TITLE-ABS ( "Primary Care" ) OR TITLE-ABS ( "Family Medicine" ) OR TITLE-ABS ( "Primary Health care" ) ) AND PUBYEAR > 2012 AND PUBYEAR < 2025 | 779 |

## Pubmed

| Search number | Query | Results |
| --- | --- | --- |
| 1 | referral*[Title] | 23,274 |
| 2 | Improve*[Title/Abstract] OR Intervent*[Title/Abstract] OR Innovat*[Title/Abstract] | 4,846,455 |
| 3 | "General Practice*"[Title/Abstract] OR "Family Practice*"[Title/Abstract] OR "Primary Care*"[Title/Abstract] OR "Family Medicine*"[Title/Abstract] OR "Primary Health Care*"[Title/Abstract] | 247,786 |
| 4 | #1 AND #2 AND #3 | 1,177 |
| 5 | #4 AND ("2013/01/01"[Date - Publication] : "2024/12/31"[Date - Publication]) | 806 |
