# Supplementary material for: Mapping the literature on improving primary-to-secondary care referrals through the lens of the Quintuple Aim framework for healthcare improvement: a scoping review
Source: BMJ Open. 2026 Aug 3;16(8):e105430. doi: 10.1136/bmjopen-2025-105430 (PMC13435927; doi:10.1136/bmjopen-2025-105430)
Supplement: Supplementary file 4 [file bmjopen-16-8-s003.docx]

## Guide for decision-Eligibility

## Inclusion Criteria

| **Criteria** | **Determinant** |
| --- | --- |
| Concept | Improvements of the referral process from primary to secondary care |
| Context | Primary Health Care |
| Population | Anyone affected by the improvement: stakeholders e.g., administrators, health care professionals, patients and next-of-kins |

### Concept

The concept is studies that focus on improvements of the referral processes, including management, guidelines, criteria, and outcomes etc. The central theme of this review is the exploration of interventions and improvements within the referral process. This includes:

- Innovative strategies and interventions aimed at enhancing the efficiency, effectiveness, and quality of the referral process from primary to secondary care.
- Evaluative studies that assess the impact of these improvements on the healthcare delivery system, healthcare professionals' practices, and patient care outcomes.

### Context

### The review is set within the context of Primary Health Care, where the initial patient evaluation occurs, and referrals to secondary or tertiary care are initiated. The context will specifically consider:

### Studies conducted in primary care settings such as general practices or family practices.

- Research focusing on the referral mechanisms within these primary care settings and their interface with secondary and tertiary care facilities.

### Population

This scoping review will encompass studies involving stakeholders who are directly impacted by improvements in the referral process.

## Exlusion criteria

### Articles that doesn’t meet the inclusion criteria:

To make the decision, use the CCP above

#### Concept: Referrals

All articles that doesn’t have referral as a main concept should be excluded even if they mention referrals in the title or abstract.

#### Concept: Improvements

The article should have some kind of intervention/improvement of referrals or the referral process, if it doesn’t it should be excluded. Qualitative studies can be included if they explore an improvement.

#### Concept: “To secondary care”

This can be a difficult one I have realized.

Things that in Swedish context are part of the primary care can be viewed as secondary care in other health care settings, things like physical or psychological therapy, radiology etc. My suggestion is therefore to include them or to discuss them in the team.

Things that are obviously not to primary health care should clearly be excluded, like social services, to other primary care settings or from secondary to primary care.

#### Context: Primary care

If the article not have a primary health care context it should be excluded. This can however be difficult to decide because it’s not always clear what IS primary care. According to my definition above primary care is GP och family practice and things like emergency department, pediatrics etc should be excluded.

#### Population

I haven’t found any articles that should be excluded because of population.

### Other exlusion criteria:

#### Not peer-reviewed articles

I have found several articles which isn’t scientific peer-reviewed studies, these should of course be excluded.

#### Not in English

Not possible to make a decision.
