# Supplementary material for: Mapping the literature on improving primary-to-secondary care referrals through the lens of the Quintuple Aim framework for healthcare improvement: a scoping review
Source: BMJ Open. 2026 Aug 3;16(8):e105430. doi: 10.1136/bmjopen-2025-105430 (PMC13435927; doi:10.1136/bmjopen-2025-105430)
Supplement: Supplementary file 5 [file bmjopen-16-8-s004.docx]

**Identification of studies via databases and registers**

Records removed *before screening*:

Duplicate records automatically removed by Endnote (n = 669)

Duplicate records removed manually using Rayyan (n = 175)

Records identified from*:

Databases (n = 1743)

Pubmed (n = 641)

Scopus (n = 779)

Cinahl (n = 323)

**Identification**

Records excluded manually using Rayyan (a record can have multiple reasons for exlusion)

(n = 745)

Wrong Concept (n = 682)

Wrong Context (n = 112)

Wrong format (n = 49)

Wrong language (n = 14)

Records screened (Title/Abstract

(n = 899)

**Screening**

Reports not retrieved

PDF not found (n = 17)

Reports sought for retrieval

(n = 154)

Reports excluded (n = 63)

Wrong concept (n = 42)

Wrong context (n = 21)

Reports assessed for eligibility

(n = 148)

Studies included in review

(n = 85)

**Included**

*Consider, if feasible to do so, reporting the number of records identified from each database or register searched (rather than the total number across all databases/registers).

**If automation tools were used, indicate how many records were excluded by a human and how many were excluded by automation tools.

Source: Page MJ, et al. BMJ 2021;372:n71. doi: 10.1136/bmj.n71.

This work is licensed under CC BY 4.0. To view a copy of this license, visit <https://creativecommons.org/licenses/by/4.0/>
