# Supplementary material for: Mapping the literature on improving primary-to-secondary care referrals through the lens of the Quintuple Aim framework for healthcare improvement: a scoping review
Source: BMJ Open. 2026 Aug 3;16(8):e105430. doi: 10.1136/bmjopen-2025-105430 (PMC13435927; doi:10.1136/bmjopen-2025-105430)
Supplement: Supplementary file 6 [file bmjopen-16-8-s006.docx]

# Plain Language Summary

## Improving Referrals Between Family Doctors and Specialists

Getting referred from a family doctor (primary care) to a specialist (secondary care) is a common and important part of healthcare. However, this process doesn't always run smoothly. Problems like delays, missing information, or poor coordination can lead to worse care for patients, higher costs, and more stress for healthcare staff. It can also mean that some people don't get fair access to the specialist care they need.

Our research team looked at existing studies from the past decade (2013–2024) to understand these referral problems and the improvements that have been tried. We used a broad framework called the "Quintuple Aim" to assess them. This means we looked at how referrals affect five key areas:

- Patient experience (Is care good for patients?)
- Population health (Does it help keep communities healthy?)
- Costs (Is it affordable and efficient?)
- Healthcare worker well-being (Are doctors and nurses supported?)
- Health equity (Does everyone have fair access and outcomes?)

We found that problems at every step of the referral process – from the initial decision to refer, to sharing information, and getting an appointment – can negatively impact all five of these important healthcare goals. Many improvements have been developed, like electronic referral systems or standardized forms. These often aim to make referrals faster or more complete. However, we found much less information about how these solutions affect the well-being of healthcare staff or whether they truly help create a fairer system for all patients.

This review highlights that just making referrals more efficient is not enough. To truly improve the system, future efforts need to make sure that improvements also support healthcare workers and ensure that everyone, regardless of their background or location, can benefit equally from good referral practices.
